# Non-atrial/non-ventricular functional tricuspid valve regurgitation: a case report

**DOI:** 10.1093/ehjcr/ytag753

**Published:** 2026-10-22

**Authors:** Netra Nambiar Veetil, Nicolas Merke, Maria Hajduzcenia, Volkmar Falk, Christoph Knosalla

**Affiliations:** Deutsches Herzzentrum der Charité, Department of Cardiothoracic and Vascular Surgery, Augustenburger Platz 1, Berlin 13353, Germany; Charité-Universitätsmedizin Berlin, Corporate Member of Freie Universität Berlin and Humboldt-Universität zu Berlin, Germany, Charitéplatz 1, Berlin 10117, Germany; German Centre for Cardiovascular Research (DZHK), Partner Site Berlin, Potsdamer Straße 58, Berlin 10785, Germany; Institute of Physiology, Charité–Universitätsmedizin Berlin, Corporate Member of Freie Universität Berlin and Humboldt-Universität zu Berlin, Charitéplatz 1, Berlin 10117, Germany; Deutsches Herzzentrum der Charité, Department of Cardiothoracic and Vascular Surgery, Augustenburger Platz 1, Berlin 13353, Germany; Charité-Universitätsmedizin Berlin, Corporate Member of Freie Universität Berlin and Humboldt-Universität zu Berlin, Germany, Charitéplatz 1, Berlin 10117, Germany; Deutsches Herzzentrum der Charité, Department of Cardiothoracic and Vascular Surgery, Augustenburger Platz 1, Berlin 13353, Germany; Charité-Universitätsmedizin Berlin, Corporate Member of Freie Universität Berlin and Humboldt-Universität zu Berlin, Germany, Charitéplatz 1, Berlin 10117, Germany; Deutsches Herzzentrum der Charité, Department of Cardiothoracic and Vascular Surgery, Augustenburger Platz 1, Berlin 13353, Germany; Charité-Universitätsmedizin Berlin, Corporate Member of Freie Universität Berlin and Humboldt-Universität zu Berlin, Germany, Charitéplatz 1, Berlin 10117, Germany; German Centre for Cardiovascular Research (DZHK), Partner Site Berlin, Potsdamer Straße 58, Berlin 10785, Germany; Department of Health Science and Technology, Translational Cardiovascular Technology, LFW C 13.2, ETH Zurich, Universitätstrasse 2, Zürich 8092, Switzerland; Deutsches Herzzentrum der Charité, Department of Cardiothoracic and Vascular Surgery, Augustenburger Platz 1, Berlin 13353, Germany; Charité-Universitätsmedizin Berlin, Corporate Member of Freie Universität Berlin and Humboldt-Universität zu Berlin, Germany, Charitéplatz 1, Berlin 10117, Germany; German Centre for Cardiovascular Research (DZHK), Partner Site Berlin, Potsdamer Straße 58, Berlin 10785, Germany

**Keywords:** Functional tricuspid regurgitation, Constrictive pericarditis, Tricuspid annular motion, Pericardial tethering, Right-sided heart failure, Multimodality imaging, Case report

## Abstract

**Background:**

Functional tricuspid regurgitation (fTR) is usually caused by annular dilation and leaflet tethering secondary to right atrial (RA) or right ventricular (RV) remodelling. Rarely, extrinsic factors may impair annular motion.

**Case summary:**

We report a 83-year-old woman with constrictive pericarditis who presented with right-sided heart failure and moderate tricuspid regurgitation. Multimodality imaging revealed a thickened pericardium, preserved RA and RV size, and a non-dilated, saddle-shaped annulus with markedly reduced motion. Following pericardiectomy, tricuspid annular mobility normalized, right-sided pressures decreased, and TR improved from moderate to mild. We introduce the term **non-atrial/non-ventricular functional tricuspid regurgitation** to describe this rare mechanism of isolated annular dysfunction caused by pericardial tethering.

**Discussion:**

This case highlights a novel, distinct, and reversible form of fTR caused by pericardial constraint rather than chamber remodelling. We propose the term **non-atrial/non-ventricular fTR** to describe this mechanism. Recognizing this entity may guide surgical decision-making and avoid unnecessary tricuspid valve intervention.

Learning points

- Functional tricuspid regurgitation can occur without atrial or ventricular dilation due to annular tethering from a constrictive pericardium.
- This form of tricuspid regurgitation may be reversible, resolving after pericardiectomy without valve intervention.
- Recognizing this distinct mechanism may improve decision-making for tricuspid valve management in constrictive pericarditis.

## Introduction

Functional tricuspid regurgitation (fTR) accounts for 70%–90% of all tricuspid regurgitation cases and typically arises secondary to tricuspid annular dilation.^1–4^ This dilation most commonly reflects right ventricular (RV) or right atrial (RA) remodelling in the context of left-sided heart disease, chronic atrial fibrillation, or pulmonary hypertension. Less frequent causes include conditions such as hyperthyroidism and pulmonic valve stenosis, which alter right-sided loading conditions through volume or pressure changes. Resulting annular enlargement and geometric distortion of the tricuspid valve apparatus lead to progressive leaflet malcoaptation despite structurally normal leaflets.

We report a rare case of fTR due to pericardial constriction in the absence of RA or RV dilation or dysfunction. This illustrates an underrecognized mechanism of functional tricuspid regurgitation in which external pericardial constraint, rather than intrinsic chamber remodelling, impairs valve competence. Recognition of such alternative mechanisms is clinically important, as reliance on atrial or ventricular dilation alone may lead to misclassification and delayed diagnosis. In particular, pericardial constriction represents a potentially reversible cause of fTR that may be overlooked when assessment is limited to conventional structural criteria. This case therefore underscores the need to consider pericardial disease in the differential diagnosis of isolated TR and right-sided heart failure.

## Summary figure

**Figure:**
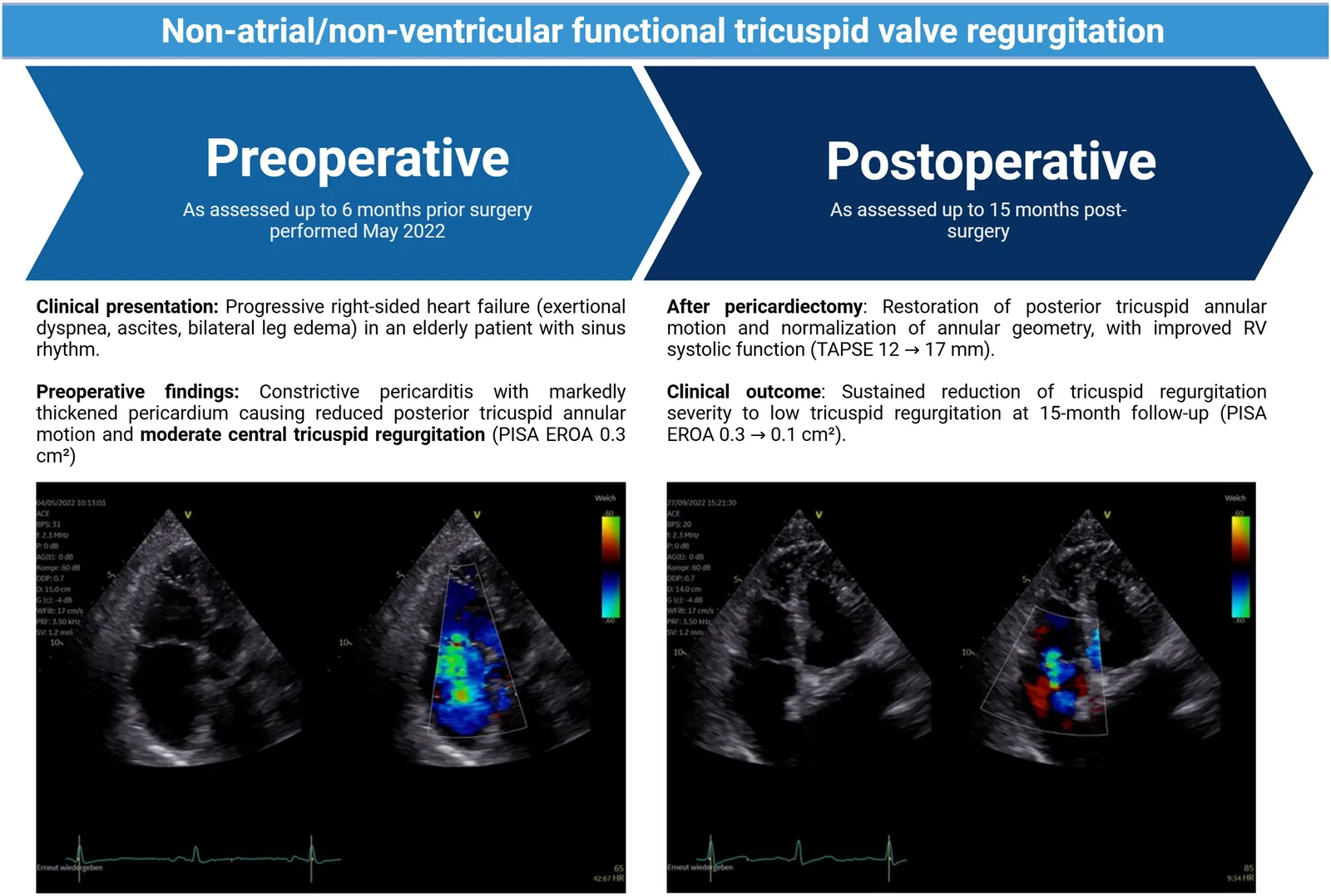


## Case presentation

An 83-year-old woman with NYHA class III symptoms was referred for evaluation of progressive exertional dyspnoea, ascites, and bilateral leg oedema. Despite a history of atrial fibrillation, electrocardiography demonstrated normal sinus rhythm pre- and postoperatively. Transthoracic echocardiography, confirmed by electrocardiography (ECG)-gated cardiac computed tomography (*Figure 1*), showed a markedly thickened pericardium with exaggerated ventricular interdependence, raising strong suspicion for constrictive pericarditis. Constriction was associated with reduced motion of the posterior tricuspid annulus, resulting in moderate central TR with a PISA-derived effective regurgitant orifice area (EROA) of 0.3 cm^2^ (*Figure 2A*, Supplementary material online, *Figure S1A*).

**Figure 1:**
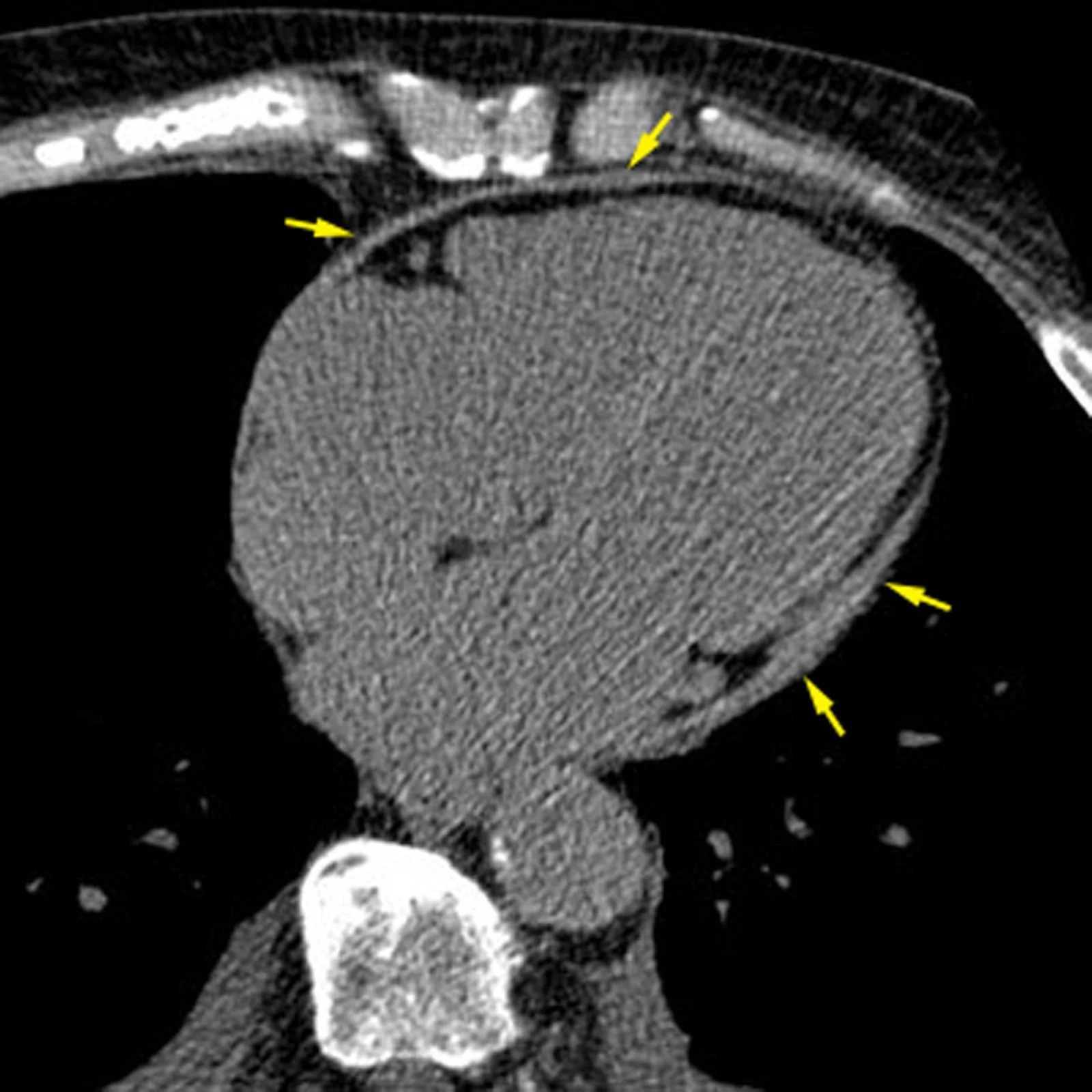
ECG-gated cardiac computed tomography shows circumferential thickening of the pericardium (marked by yellow arrows).

**Figure 2:**
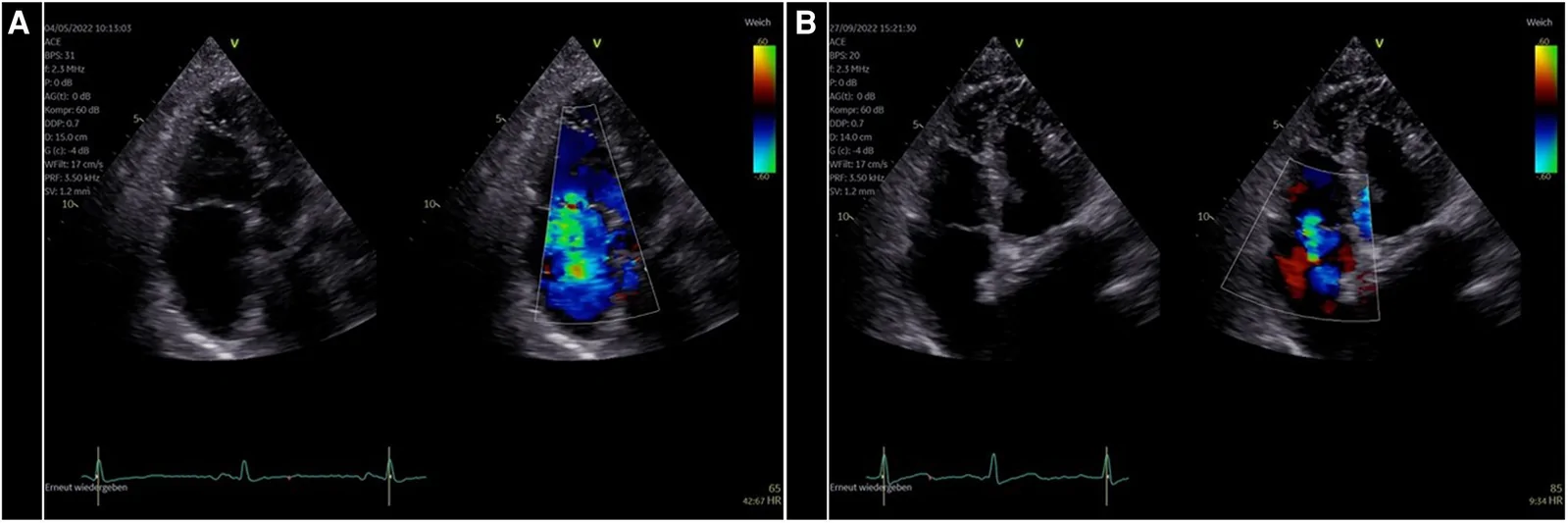
Transthoracic evaluation in a case of constrictive pericarditis shows moderate tricuspid valve regurgitation (green-yellow jet) in the presence of normal right atrial and ventricular function and size, with normal tricuspid valve leaflets in size and morphology (*A*), and mild tricuspid valve regurgitation (green-yellow jet) after pericardiectomy (*B*).

In contrast to typical fTR—characterized by annular dilation and geometric remodelling^5,6^ —annular geometry was preserved. 3D echocardiography confirmed a non-planar, saddle-shaped annulus without dilation and preserved global right ventricular systolic function (3D ejection fraction), despite reduced longitudinal annular excursion (tricuspid annular plane systolic excursion [TAPSE] 12 mm) (*Figure 3A*, Supplementary material online, *Figure S2A*). Right ventricular free-wall longitudinal strain was preserved (−24%), indicating preserved myocardial deformation in the presence of reduced longitudinal annular motion (see Supplementary material online, *Figure S3A*). Right atrial size was normal preoperatively (60 mL; indexed 31 mL/m^2^), with reduction postoperatively (35 mL; 20 mL/m^2^) (*Figure 4*), while right ventricular dimensions remained within normal limits throughout (basal diameter 26 mm vs. 31 mm; mid-ventricular diameter 26 mm in both assessments) (see Supplementary material online, *Figure S4*). Right atrial strain analysis showed impaired reservoir and contractile function initially, with improvement following pericardiectomy, consistent with reversible functional atrial impairment (see Supplementary material online, *Figure S5*).^8^ Quantification of tricuspid regurgitation and right heart dimensions was performed in accordance with current guideline recommendations for valvular heart disease.^4,9^

**Figure 3:**
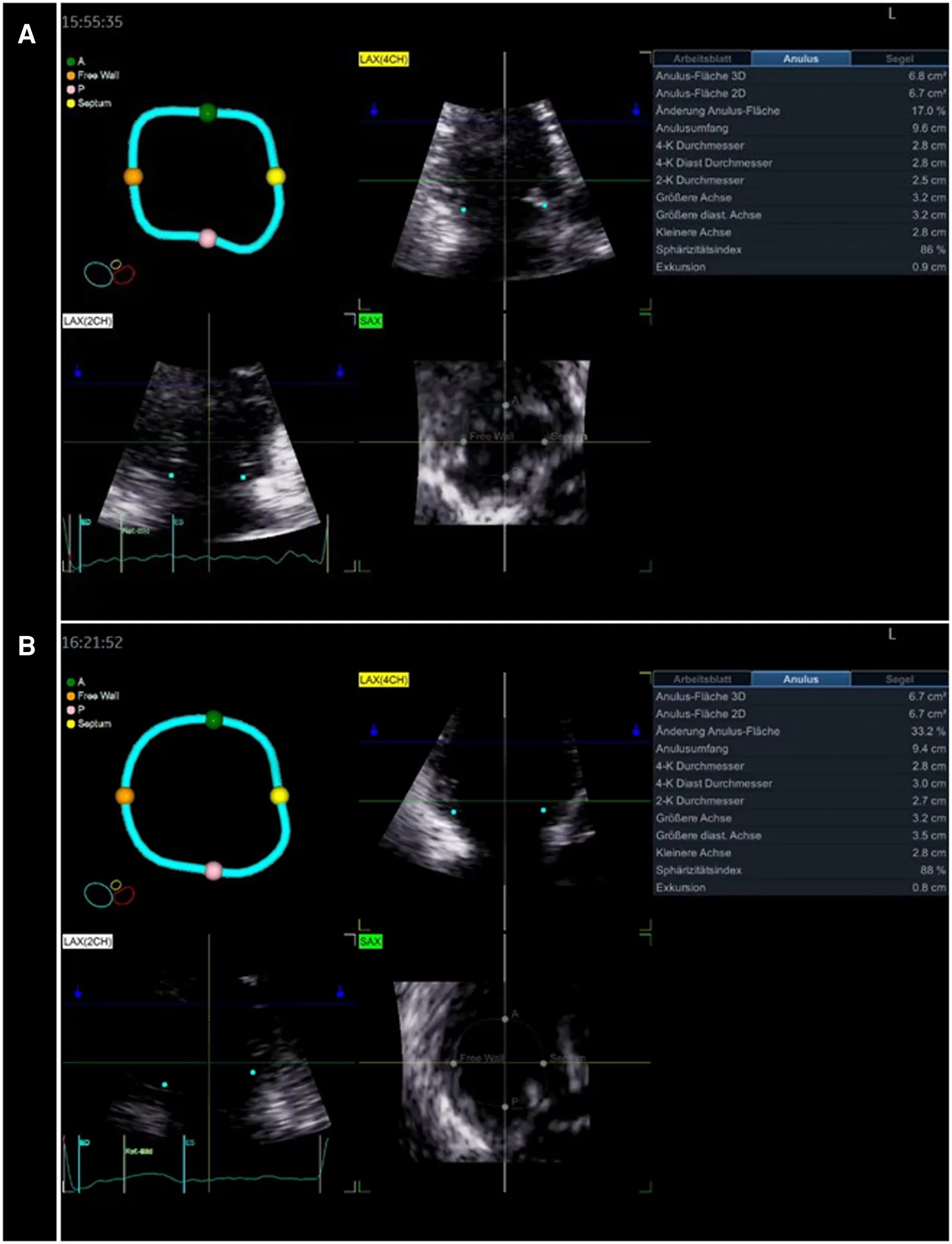
Analysis of the geometry and motion of the tricuspid annulus using a dedicated software package for 3DE analysis of the tricuspid valve (TV) (4D Auto TVQ, Version 204; GE Vingmed, Horten, Norway^14^) before surgical pericardium removal shows constrictive TV annular movement with reduced motion of the fixed posterior annulus, resulting in an asynchronous and reduced motion pattern of the annulus (*A*). After pericardial removal, 3 DE analysis revealed improved motion of the posterior tricuspid valve annulus and normalization of tricuspid shape (*B*). The methodology used to acquire the 3DE TV and the analysis workflow used by the 4D AutoTVQ software have recently been described in detail elsewhere by D. Muraru, L. Badano, and F Veronesi.^7^

**Figure 4:**
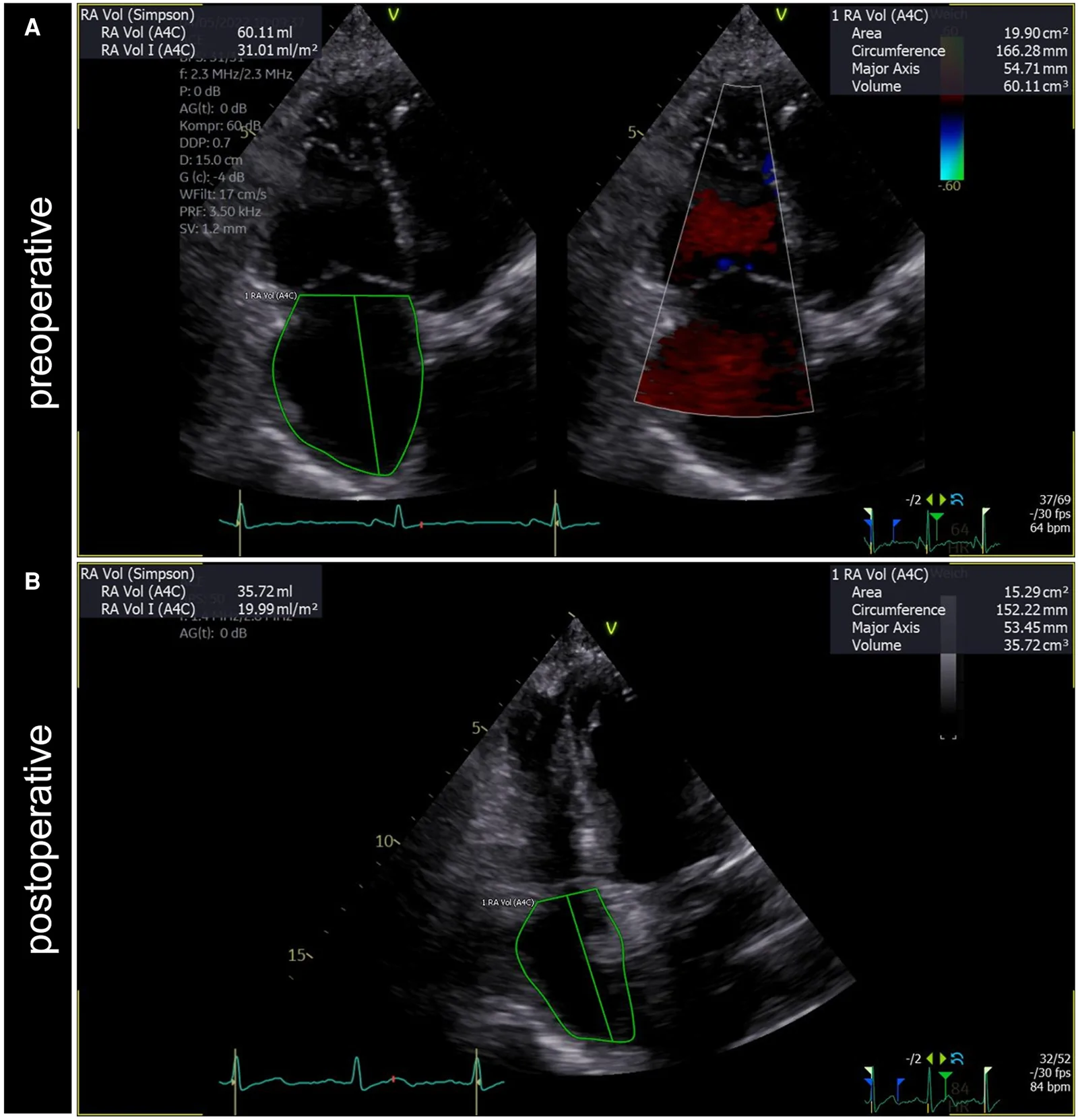
Echocardiographic assessment of right atrial size pre- and postoperatively. Representative echocardiographic images demonstrating right atrial size before and after surgery. Preoperatively, right atrial dimensions corresponded to a normal right atrial volume of 60 mL (indexed 31 mL/m^2^). Postoperatively, a reduction in right atrial size was observed, with a volume of 35 mL (indexed 20 mL/m^2^), consistent with reverse atrial remodelling following surgical intervention.

Right heart catheterization demonstrated diastolic pressure equalization (20 mmHg) with a characteristic ‘square root sign’—early diastolic dip followed by a rapid rise and plateau in ventricular pressures—confirming constrictive pericarditis. Mean pulmonary artery pressure was 29 mmHg with a pulmonary capillary wedge pressure of 24 mmHg. Coronary angiography showed no significant stenosis. Given symptomatic heart failure (NYHA class III) and haemodynamically confirmed constrictive physiology, the patient was referred for surgery and subsequently underwent successful surgical pericardiectomy. Inflammatory markers were unremarkable (white blood cell count: 8.83 × 10^9^/L, C-reactive protein: 19 mg/L), arguing against acute active inflammation, making anti-inflammatory therapy unlikely to provide clinical benefit in this advanced stage of disease. Notably, pericardial calcification was absent but is not required for diagnosis or surgical indication, which primarily rely on clinical presentation and invasive haemodynamic findings, as reflected in current guideline recommendations.

Postoperatively, TAPSE improved (12 to 17 mm) along with posterior annular motion, with restoration of annular dynamics (*Figure 3B*, *Table 1*, Supplementary material online, *Figure S2B*). At 15-month follow-up, TR severity decreased significantly, with EROA reduced to 0.1 cm^2^ (*Figure 2B*, Supplementary material online, *Figure S1B*).

**Table 1:** Echocardiographic parameters before and after pericardiectomy

*Parameter*	*Preoperative*	*Postoperative*
*RA volume (ml)*	60	35
*Right Atrial Volume Index (RAVI, mL/m^2^)*	31	20
*RA reservoir strain (%)*	10.5	19.9
*RA conduit strain (%)*	−6.4	−10.8
*RA contractile strain (%)*	−4.1	−9.0
*RV free-wall longitudinal strain (%)*	−24%	−25%
*RV basal diameter (RVEDD, mm)*	26	31
*RV mid-ventricular diameter (RVEDD, mm)*	26	26
*RV ejection fraction—3D (%)*	49	52
*TAPSE (mm)*	12	17
*Major axis of TV annulus (mm)*	32	35
*Projected tricuspid annular area changes (%)*	17	33

The improvement of the restricted movement of the tricuspid annulus is best shown by the increased TV annulus major axis from 32 to 35 mm and the increase of the per cent change of the projected tricuspid annulus area from 17% to 33% after pericardiectomy.

RA, right atrium; RV, right ventricle; TAPSE, tricuspid annular plane systolic excursion; TV, tricuspid valve.

This case highlights a distinct mechanism of fTR due to extrinsic annular constraint from pericardial thickening rather than intrinsic chamber remodelling. Unlike conventional atrial or ventricular fTR—often associated with persistent or progressive TR after pericardiectomy^5^—this form appears reversible following surgical decompression, with potential implications for timing and selection of surgical intervention.

## Discussion

We present a rare and, to our knowledge, previously unreported form of non-atrial/non-ventricular fTR, caused by pericardial constraint in the absence of RA or RV remodelling. Current classifications of fTR emphasize annular dilation, leaflet tethering, and chamber remodelling—typically in the context of left-sided heart disease, pulmonary hypertension, or atrial fibrillation,^10^ resulting in a dilated, planar annulus and progressive RV enlargement.^1^ In contrast, this case demonstrates that pericardial constraint alone can impair annular motion and lead to clinically relevant regurgitation despite preserved right heart geometry. The discrepancy between reduced TAPSE and preserved RV ejection fraction and free-wall strain reflects the known dissociation between longitudinal annular motion and myocardial deformation in constrictive pericardial physiology. Pericardial restraint limits annular excursion and global cardiac translation, while intrinsic myocardial contractility remains preserved, as evidenced by normal strain and 3D RV function. Accordingly, displacement-based parameters such as TAPSE are more susceptible to external constraint, whereas strain more accurately reflects myocardial deformation.^11^ These findings support a predominantly extrinsic, rather than myocardial, mechanism of dysfunction.

Right atrial strain analysis further supports a reversible, load-dependent impairment. Despite normal RA size, strain was initially reduced and improved following pericardiectomy, consistent with functional limitation due to pericardial constraint rather than intrinsic atrial disease. Although pulmonary artery pressure was elevated preoperatively, the absence of right atrial or right ventricular remodelling, together with preserved RV systolic function, argues against chronic pressure overload as the primary mechanism of tricuspid regurgitation. Importantly, surgical intervention was guided by symptomatic, haemodynamically confirmed constrictive pericarditis, not by TR severity.

To our knowledge, no prior reports have described pericardial constriction presenting with secondary fTR due to extrinsic annular constraint in the absence of right-sided chamber remodelling—making this phenotype unique. Prior reports instead describe (i) severe TR mimicking constrictive physiology in the setting of annular dilation and chamber enlargement^12^ and (ii) development or progression of TR after pericardiectomy due to RV dilation, which is associated with adverse outcomes.^13^ In large cohorts (including >500 patients), even mild TR preoperatively is linked to significantly higher mortality after pericardiectomy (hazard ratio ∼1.6–2.3), independent of RV function.^13^ Our case diverges sharply: our patient demonstrated improvement in annular mobility and geometry, increased TAPSE, recovery of right atrial function, and reduction in PISA-derived EROA from 0.3 to 0.1 cm^2^ following surgery.

These findings define a distinct mechanistic subtype of fTR driven by extrinsic pericardial constraint in the absence of right atrial or right ventricular remodelling, which is not captured in current classification frameworks. Unlike remodelling-mediated fTR, this form appears reversible with surgical decompression. Recognition of this mechanism has important clinical implications, as it identifies a potentially reversible cause of TR and supports evaluation for constrictive pericarditis in patients with otherwise unexplained TR and preserved right heart geometry. Surgical decision-making should therefore prioritize the haemodynamic severity of constriction rather than TR severity alone, as valve intervention may not be required.

Further studies, including additional cases with longitudinal imaging and haemodynamic correlation, are needed to validate this subtype, define diagnostic criteria, and clarify implications for surgical planning.

## Lead author biography


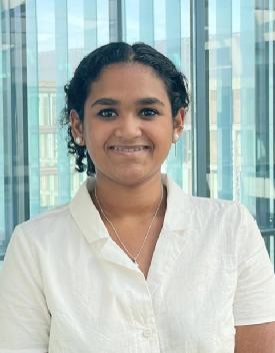
 Netra Nambiar Veetil, MD, is a resident physician in cardiac surgery at the German Heart at Charité University Hospital in Berlin, Germany. She graduated from medical school in Germany and has clinical experience in perioperative and postoperative care of patients with complex cardiac disease. Her research interests focus on right heart pathology, valvular heart disease, pulmonary hypertension, and cardiopulmonary interactions. In 2025, she received the Young Investigator Award for Clinical Cardiovascular Research from the German Society of Cardiology. She has presented her work at several international scientific meetings and is engaged in translational and clinical cardiovascular research.

## Supplementary Material

ytag753_Supplementary_Data

## Data Availability

The data underlying this article are available in the article and in its online Supplementary material.
