# Supplementary material for: Non-atrial/non-ventricular functional tricuspid valve regurgitation: a case report
Source: Eur Heart J Case Rep. 2026 Oct 22;10(11):ytag753. doi: 10.1093/ehjcr/ytag753 (PMC13738075; doi:10.1093/ehjcr/ytag753)
Supplement: Supplementary file 1 [file ytag753_supplementary_data.zip › Supplementary Figure Legends.docx]

**Supplementary Material :**

**Figure S1:** Corresponding videos to Figure 2, panels (a) and (b)

**Alternative Text Figure S1**: Echocardiographic videos corresponding to Figure 2, showing tricuspid regurgitation before and after pericardiectomy.

**Figure S2:** Corresponding videos to Figure 3, panels (a) and (b)

**Alternative Text Figure S2**: Three-dimensional echocardiographic videos corresponding to Figure 3, showing tricuspid annular motion before and after pericardiectomy.

**Figure S3**: Pre- and postoperative assessment of right ventricular free-wall longitudinal strain.

**Alternative Text Figure S3**: Pre- and postoperative echocardiographic assessment of right ventricular free-wall longitudinal strain, showing preserved right ventricular function before and after pericardiectomy.

**Figure S4**: Three-dimensional echocardiographic assessment of right ventricular function postoperatively

**Alternative Text Figure S4**: Three-dimensional echocardiographic assessment of right ventricular function after pericardiectomy, showing preserved right ventricular systolic function.

**Figure S5:** Right atrial strain analysis performed using vendor-independent speckle-tracking software (TomTec Imaging Systems), showing reservoir (RASr), conduit (RAScd), and contractile strain (RASct) at baseline and during postoperative follow-up.

**Alternative Text Figure S5**: Right atrial strain analysis showing reservoir (RASr), conduit (RAScd), and contractile (RASct) strain at baseline and during postoperative follow-up.
